# Blue phase liquid crystal: strategies for phase stabilization and device development

**DOI:** 10.1088/1468-6996/16/3/033501

**Published:** 2015-05-05

**Authors:** M D Asiqur Rahman, Suhana Mohd Said, S Balamurugan

**Affiliations:** Solid State Nanodevices Laboratory, Department of Electrical Engineering, Faculty of Engineering, University of Malaya, 50603 Kuala Lumpur, Malaysia

**Keywords:** blue phase liquid crystal, Kerr effect, electrode architecture, polymer stabilization, nanoparticles addition

## Abstract

The blue phase liquid crystal (BPLC) is a highly ordered liquid crystal (LC) phase found very close to the LC–isotropic transition. The BPLC has demonstrated potential in next-generation display and photonic technology due to its exceptional properties such as sub-millisecond response time and wide viewing angle. However, BPLC is stable in a very small temperature range (0.5–1 °C) and its driving voltage is very high (∼100 V). To overcome these challenges recent research has focused on solutions which incorporate polymers or nanoparticles into the blue phase to widen the temperature range from around few °C to potentially more than 60 °C. In order to reduce the driving voltage, strategies have been attempted by modifying the device structure by introducing protrusion or corrugated electrodes and vertical field switching mechanism has been proposed. In this paper the effectiveness of the proposed solution will be discussed, in order to assess the potential of BPLC in display technology and beyond.

## 1. Introduction

Liquid crystal (LC) is a state matter intermediate between the solid and liquid phases; it was first discovered by Reinitzer in 1888 [1]. The unique properties of LCs, such as optical anisotropy and response to electric field, made them popular materials for electronic displays. Several subphases exist within the LC phase, such as smectic, nematic and cholesteric subphases, which were described by Friedel in 1922 [2]. In 1888, the discovery of LC by Reinitzer [1, 3] involved a unique LC subphase, called the blue phase (BP), which can be found as one cools down the LC from the isotropic phase [4–10]. The BP structure is of chiral nematic LC that is highly twisted. The temperature window of BP is very narrow (0.5–1 °C) and can only be observed between the isotropic and helical phases of cholesteric LC [11]. The BP can be divided into three categories (BPI, BPII and BPIII) depending on the chirality of the LC. The BPIII phase has an almost similar structure to the isotropic phase [12], whereas BPI and BPII are made out of double-twist cylinders packed in cubic lattices [13].

The most noticeable characteristic of BPs is that it demonstrates selective reflection of incident light. Its name is derived from the historical observation of the first BPs studied, which exhibited blue color when observed under polarizing optical microscope. However, the BP does not necessarily show blue coloration [14], as coloration depends on the pitch of the LC within the BP. The BPs have potential to be used in fast light modulators or tunable photonic crystals. However, due to the narrow range of temperature where the BP exists, it is still far from being realized in practical applications [15].

To date, considerable research effort has been expended to widen the temperature window of blue phase liquid crystals (BPLCs), in order to render it viable for commercial applications such as displays and photonic technology. Two main strategies are through the addition of polymer or nanoparticles (NPs). For example, the widening of the temperature range of BPs can be achieved through composite systems which consist of polymers and low molecular weight LC. Kikuchi *et al* [15] succeeded in expanding the BP temperature range by more than 60 °C through the use of such composite systems. Another way of widening the temperature range of BPLCs is by incorporating NPs, where the NPs accumulate in the lattice disclination lines of the BP and stabilize the overall cholesteric blue structure. Studies from systematic high-resolution have found that the mixing of surface-functionalized NPs with LC can lead in the stabilization of BPIII in a wide temperature window [16].

A hybrid BPLC system was reported by Dierking *et al* [17] and later Wang *et al* [18] where a combination of polymer and NPs were used to stabilize and extend the temperature range of the BP. By dispersing the NPs (of diameter ∼30 nm) at a concentration of about 0.5 wt% into the polymer-stabilized blue phase (PSBP) system comprising 5.0 wt% polymers (trimethylolpropane triacrylate; TMPTA) and bifunctional monomer (C6M), the mixture showed an increase in the BP range with a maximum of 41.2 °C for ZnS-doped PSBP and 45.9 °C for ferroelectric BaTiO_3_-doped PSBP. It is expected that the hybrid (polymer-stabilized NP-doped) method stabilizes the disclination lattice of BPI by both the NPs and polymer chains which is more effective and efficient than by single polymer stabilization or NP dispersion [18]. Moreover, a numerical study and advance concept in material design was discussed briefly by Yoshizawa for stabilizing the BP system [19].

The use of LC in displays was first realized in 1968. George Heilmeier invented a display which was based on the dynamic scattering effect [20–22]. This gave rise to a popularity in black and white displays in calculators and watches in 1970 [23]. In 1971, Schadt, Helfrich and Fergason invented independently at almost the same time the twisted nematic (TN) mode [24–29], which was an important milestone in the development of liquid crystal display (LCD) technology. In 1988, Washizuka and Takeda of Sharp Corporation introduced addressation of LCD pixels using thin film transistor arrays, which enabled fast switching for a large array of pixels. This resulted in the active-matrix full-color full motion LCD, which is the precursor to color LCDs in use today [30].

PSBPLC have been proposed as the next generation LCDs [31, 32] due to their advantages of sub-millisecond response time (ten times faster than conventional nematic) [33, 34], lack of alignment layer (typically needed) to guide the alignment of LC, and an inherent wide viewing angle characteristic. The BPLC is especially attractive for applications such as large-screen LCDs, as the BP is optically isotropic unlike the nematic phase, therefore providing wide viewing angle and excellent dark state without any compensation films [35]. The Kerr effect arises during the isotropic to BP transition which induces a change in birefringence. It is a nonlinear (quadratic) optical effect, which arises during the reorientation of the LC director upon application of an electric field *E*. It is expressed as *Δn* = *λKE*^2^, where *λ* is wavelength and *K* is the Kerr constant [36–38].

However, there are still some problems in the commercialization of BP devices: (1) the operating voltage is as high as several tens of volts, which limit the use of thin-film transistor as the addressing element for BPLCDs; (2) the low transmittance (∼75%) caused by the low aperture ratio due to the presence of strip electrodes for driving the switching voltage; (3) the poor dark state which forms after a few driving frames, which is induced by the residual birefringence [39] and decreases the contrast ratio [40]; and (4) the switching hysteresis [41] induced by the chirality [42] or polymer network [43] makes consistent gray levels difficult to obtain [44].

There are several solutions that have been identified to lower the operating voltage [45–47] and make hysteresis free BPLC devices [39, 48–51]. For example, modifications to the device structure have been proposed, such as the planar in plane switching (IPS) structure [52], interdigitated indium tin oxide (ITO) electrodes [53], the protrusion electrode structure [54], periodic corrugated electrodes [36], double-side in plane switching (DS-IPS) [44], and vertical field switching (VFS) [55]. Recently, a BPLC material with a large Kerr constant (*K* ∼ 33.1 nm V^−2^ at *λ* = 514 nm) was developed, which successfully reduced the driving voltage from 100 to ∼8.4 V [56]. Secondly, wall-shaped and protrusion electrodes have been shown to reduce the operating voltage due to the thick LC layer being driven by the thin electrodes. Unfortunately, the low aperture ratio resulted in a very low transmittance and the fabrication of the electrodes is very difficult. The effect of different shapes of protrusion electrodes have also been investigated extensively but the cost of fabrication limits its practical adoption. An ideal display specification for BPLCs would include a simple electrode architecture for ease of fabrication, coupled with low operating voltage (<10 V) and high transmittance (>90%).

This paper will address the various issues associated with the practical commercialization of BPLC devices, which relies on a wide BP temperature range and also efficient device architecture. First, the background of the physics and chemistry of BPs will be introduced. Then, the strategies for widening the BP temperature range through composite mixtures (NPs or polymer) will be discussed, followed by novel device structures for BPLC devices. Finally an evaluation of the potential applications for BPLCs will be presented, in order to summarize the commercial viability of BPLCs.

## 2. What are BPs?

### 2.1. Fundamental properties of BPs

In the study of LCs, BPs are self-organized structures. The BP can be observed in many cholesteric LCs, as there are one or more intermediate phases between the transition of regular helical cholesteric phase and the isotropic phase [57]. These are the cholesteric ‘BPs’, which are thermodynamically stable for a very narrow temperature range, usually less than 1 °C [58]. Although the BPs were first discovery by Reinitzer in 1888 [1], serious investigation on BPs only started in the 1960s, when Saupe [59] found out that BPs were not only optically isotropic, but also exhibited unusually strong optical activity. The optical isotropy of the BP in its unswitched state is due to its twisted (cholesteric) direction arrangement. As an example, normally under a crossed polarizers arrangement, light cannot pass through the BP when there is no external voltage, due to the helical twisting of the directors. However, the application of voltage results in deformation of the director twist, which then allows light to pass through the polarizers. Based on his observation, Saupe proposed that the BPs possess a cubic superstructure, hypotheses which set the groundwork for BPLC studies. In 1973, Coates and Gray proposed the name, ‘BPs’, to define this unusual state and they treated the BP as a LC sub-phase [60]. The BP further characterized by Bragg scattering experiment [61–64] and single crystal structure [65, 66], to identify BP 1 (BPI) and BP 2 (BPII) which are body centered and simple cubic structures, respectively. Later, much experimental and theoretical effort was expended in the study of BPLCs such as introducing polymers or NPs or dispersion of gold nanorods to widen the temperature range, by changing the cell structure or increasing the Kerr constant to reduce the driving voltage [23, 62, 67].

### 2.2. The structure of BPs

**Figure 1.**
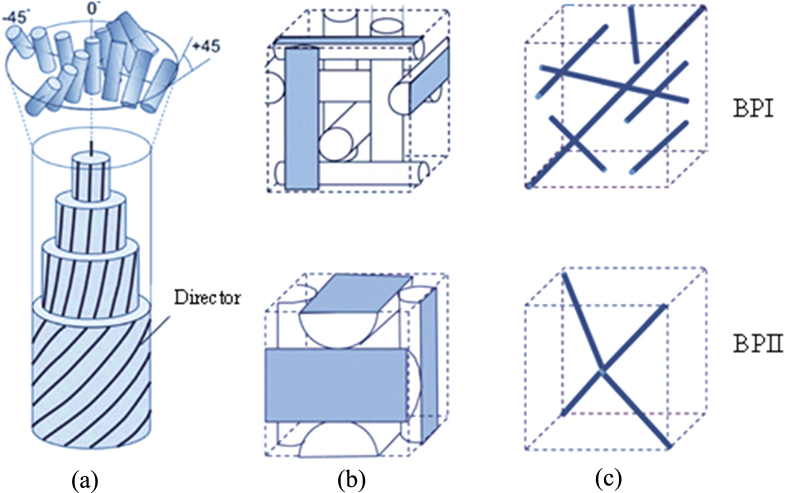
Structure of blue phases [4]. Reproduced with permission from Kikuchi H 2008 Liquid crystalline blue phases *Liquid Crystalline Functional Assemblies and Their Supramolecular Structures* vol 128 ed T Kato (Berlin: Springer) pp 99–117, with kind permission from Springer Science and Business Media.

Figure 1(a) visualizes the local director field of the double-twist cylinder which formed within such a stable area. At the central axis of the cylinder the director is vertical and it twists continuously along any radial direction. Figure 1(b) shows the double-twist cylinder in the body centered cubic unit cell of BPI and the simple cubic unit cell of BPII, respectively. And figure 1(c) shows the BPI and BPII unit cells contain disclination lines resulting from the points of intersection between the different directions of director within the cubic cell.

**Figure 2.**
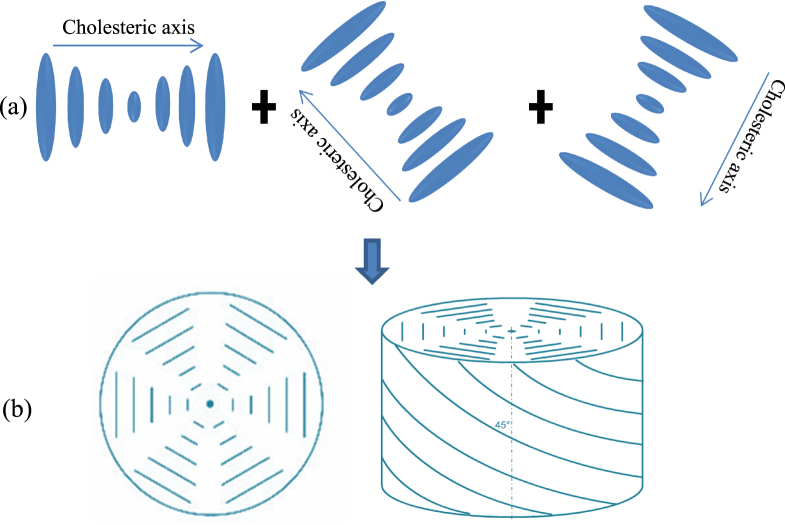
Schematic diagram of the planar hexagonal blue phase. (Author: Panjasan, source: http://en.wikipedia.org/wiki/Blue_phase_mode_LCD.)

The structure of the BP may be further understood by taking a cholesteric structure as a point of reference. For a cholesteric (chiral) LC, the director twists to form a helix over a certain pitch, *P*. In contrast the BP takes on a double-twist formation [68], by superimposing three elements of cholesteric LC but with their helical axes rotated from one to another by 120° as shown in figure 2(a). From figure 2(b), it can be seen to from a local double-twist structure. This 120° rotation between the three cholesteric helices also impose a hexagonal symmetry, which focus a unit cell generates a two dimensional lattice. The point of intersection between these three helical axes form an *s* = −1/2 disclination, i.e. the structure is locally frustrated at these intersections [69].

**Figure 3.**
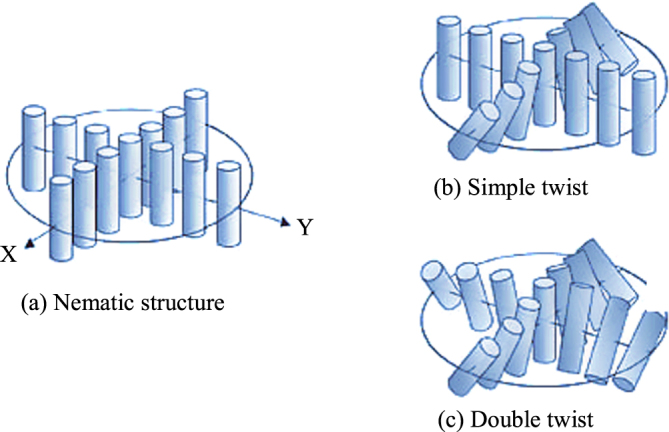
Differences between single-twist and double-twist structure [4]. Reproduced with permission from Kikuchi H 2008 Liquid crystalline blue phases *Liquid Crystalline Functional Assemblies and Their Supramolecular Structures* vol 128 ed T Kato (Berlin: Springer) pp 99–117, with kind permission from Springer Science and Business Media.

Figure 3 further elaborates on the difference between the double-twist, and the nematic and simple twist structures. Figure 3(c) shows the structure of the molecule that twisting in all lateral directions which is called a double-twist. As it spreads radially, the double-twist becomes weaker which allows complete formation of double twisting only at the center molecules and its surroundings. Since twisting is allowed in all lateral directions, the molecules are more stable than that in the case of a simple twist at and around the center. Defects occur if the double-twist cylinder expands to a broader area. As mentioned previously, a double-twist structure cannot be an introductory structure that continuously filled a space. However the BP maintains this inconsistency as it is built upon such a double-twisted cylinder structure [70].

**Figure 4.**
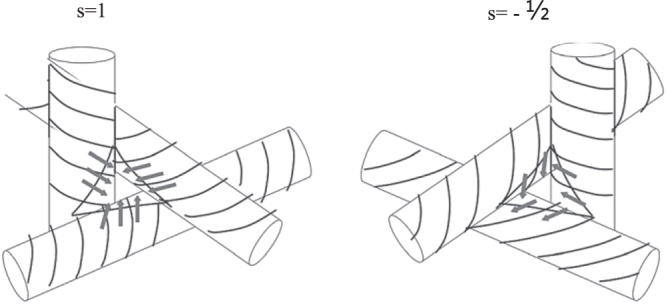
The intersect points of three double-twist cylinders [72]. Reprinted figure with permission from Sethna J P 1985 *Phys. Rev.* B **31** 6278–97, copyright 1985 by the American Physical Society.

Figure 4 illustrates a close up of the void region in the disclination lines. A key solution to a wide temperature stabilization range of the BP is enabled through filling of these disclination regions with NPs or polymer materials. Disclination lines (singularities of the director field) emerge because the void of the unit cell cannot be filled continuously by the double-twist cylinder [11, 71]. In the area of the disclination lines, the orientational order parameter vanishes into the isotropic phase. Defects occur at the points where the cylinders are in contact as illustrated in figure 4. Due to this defect, the structure of the BP is less stable and is limited to a narrow temperature range. It is also due to this fact that the BP exists in the temperature range closest to the isotropic phase [69].

BPIII is somewhat different and has an amorphous structure with the same symmetry as the isotropic fluid [11]. However, there is critical point for the phase transition from BPIII to isotropic, so that at high chirality, BPIII gradually changes to isotropic [73–75]. The director field texture and properties of BP III are not as well understood as those of the crystalline BPs [69, 76, 77].

## 3. The Kerr effect in BPLCs

The Kerr effect was discovered by John Kerr in 1875 [78]. The Kerr effect, also known as the quadratic electro-optical effect, explains that the applied electric field will realign the LC director and hence alter the refractive index [79, 80]. The sample becomes birefringent, due to this influence, with different indices of refraction for light polarized perpendicular to or parallel to the applied field. Thus, the induced birefringence, *Δn*_ind_, is given by

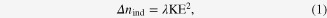
where, *λ*, *K*, and *E* represent the wavelength of the light, Kerr constant and strength of the electric field respectively. This induced birefringence causes the material to act like a wave-plate when light is incident on it in a direction perpendicular to the electric field. Equation (1) can also be written as [44]

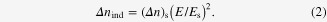
Here, (*Δn*)_s_ is the maximum induced birefringence. When the electric field *E* is higher than the saturation field *E*_s_, the induced *Δn* saturates at (*Δn*)_s_ [81]. Given the inherent anisotropic nature of LCs, the optical parameters of BPLCs are well suited to exploit the Kerr effect. To avoid induced lattice distortion, which is also known as the electrostriction effect, and resulting phase transition, a BPLC device should be operate below a critical field [82]. The critical field is defined as the peak voltage where the polymer network remains stable and the structure of the BPLC is reversible. The magnitude of this critical voltage depends on the concentration and structure of polymers employed [83]. A BPLC device will show large hysteresis and longer response time if it is driven above the critical voltage. However, in a separate investigation, Xu *et al* proposed a mechanism which reduced the hysteresis significantly and dramatically improved response time by suppressing the electrostriction effect using linear polarized UV light during the polymerization process [84]. Due to the Kerr effect, switching can be achieved with a small amount of electric field if a BPLC material contains large values of the Kerr constant [85, 86]. However, equation (2) is effective only when the applied electric field is weak. As the electric field keeps increasing, the induced birefringence will progressively saturate as indicated by the extended Kerr effect model [81]

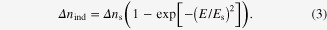


Expanding equation (3) the Kerr constant can be deduced as:

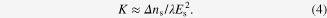


From equation (4), high *Δn*_s_ and low *E*_s_ play similarly significant roles for improving Kerr constant.

## 4. Expanding the stable temperature range of BP through composite BPLC mixtures

As mentioned previously, the temperature range for BPLC is limited to a few degrees (0.5–2 °C), which hinders the application of BP in commercial devices. Numerous attempts have been made to broaden the temperature range of the BP, such as using homogeneous mixture of LCs and NPs [16], and polymer stabilization [15]. Stabilization in the temperature range a of few degree centigrade attained for various methods for instance, using a mixture of chiral and bent-core LCs [87] and LC quaternary mixtures [88]. Kitzerow *et al* [89] formed stabilized BP structures in 1993, using reactive LC monomers, that can be photo-polymerized, which then did not demonstrate any dynamic switching behavior as all of the molecules were polymer stabilized. In 2002, Kikuchi *et al* described a non-reactive BPLC stabilized through a small amount of polymer (∼8 wt%). This polymer addition causes the cross-linking polymer network, which is selectively concentrated in the disclination cores. The temperature interval of BPLC has been expanded to more than 60 °C, containing room temperature (−13 to 52.85 °C) by stabilized the lattice structure of BP. Additionally, a fast electro-optical switching property was retained for this mixture [15]. This method has opened a new pathway for display and photonic applications [13].

### 4.1. NP induced stabilization of BP

Since 2009, the suspension of NPs in LCs has been an attractive topic for technological and scientific interest. The motivation for this interest arises from the fact that properties that have not been observed in the pure material appear as a result of the interaction between the nano-sized particles and the anisotropic host and various LC phases act as platforms to drive self-organization assisted NP assembly [90]. However, there are many approaches on mixing NPs with uniaxial anisotropic materials such as nematic LCs [91–93] and BPs [94–97]. Most recently, Yoshida *et al* reported the extension of the temperature interval of cholesteric BPs by doping gold NPs [90] and Karatairi *et al* reported the expansion of the temperature interval of BP by doping CdSe NPs [16].

Yoshida *et al* employed the gold NPs (diameter 3.7 nm), which were dispersed in the material by the sputter-doping technique. The BP-exhibiting LC material used was a mixture, shown in table 1.

**Table 1.** Nanoparticles doped as composition [90].

Materials		
Name	Type	Weight (%)
5CB	Nematic LC	46.5
JC-1041XX	Nematic LC	46.5
ISO(6OBA)2	Chiral dopant	7

**Figure 5.**
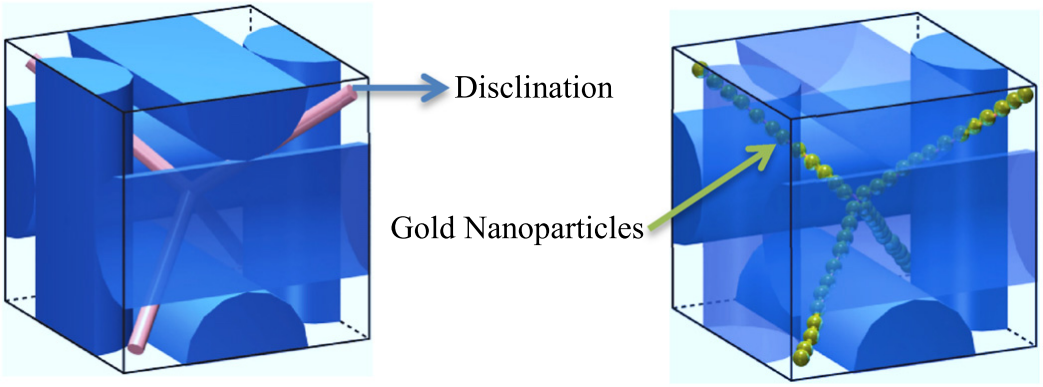
Blue phase stabilized by gold nanoparticles which trapped in the disclination lines [90]. Figure reprinted with permission from H Yoshida *et al* 2009 *Appl. Phys. Express* **2** 121501, copyright 2009 The Japan Society of Applied Physics.

It was found that with the incorporation of spherical gold NPs, the temperature range of BP expanded from 0.5 to 5 °C and decreased the clearing temperature by approximately 13 °C. This observation was attributed to a decrease in the free energy caused by self-organization-assisted NP assembly, which helped stabilize the BPLC over a larger temperature range. Ravnik *et al* reported that NPs doped in BPs were positively trapped in the disclination lines, therefore elastic interactions occur [98]. It is assumed that, at first freely moving NPs became locked in, once they met a disclination line, and so on the volume and the energy associated with the disclination lines were consequently decreased. An imaginable model of this assumption is illustrated in figure 5, where the double-twist helical structure indicated by the cylinders.

Table 2 also shows the phase transition temperature of the samples demonstrated by Yoshida *et al* successfully extended the temperature range by the addition of gold NPs.

**Table 2.** Phase transition temperature before and after nanoparticles doping [90]; (Ch—cholesteric phase and Iso—isotropic phase).

	Heating (°C)			Cooling (°C)		
Phase transition	Ch → BPI	BPI → BPII	BPII → Iso	Iso → BPII	BPPII → BPI	BPI → Ch
Pure	45	45.5	45.5	46.4	45.4	43.1
Phase transition	Ch → BP	BP → Iso		Iso → BP	BP → Ch	
NPs-doped	28.6	33.6		33.5	25.4	

**Figure 6.**
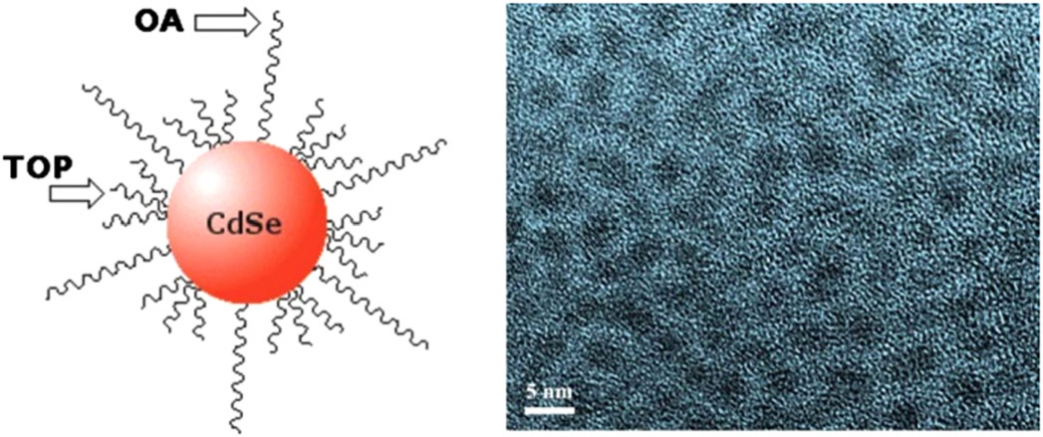
Schematic of CdSe NPs with oleyl amine (OA; left), transmission electron microscopy image of CdSe NPs (right) [16]. Reprinted figure with permission from E Karatairi *et al* 2010 *Phys. Rev*. E **81** 041703, copyright 2010 American Physical Society.

In a separate investigation, Karatairi *et al* studied the effect of adding CdSe (average diameter 3.5 nm) NPs to chiral LCs, CE8 and CE6 respectively. The size of nanocrystal and the morphology of the semiconducting NPs are shown in figure 6.

**Figure 7.**
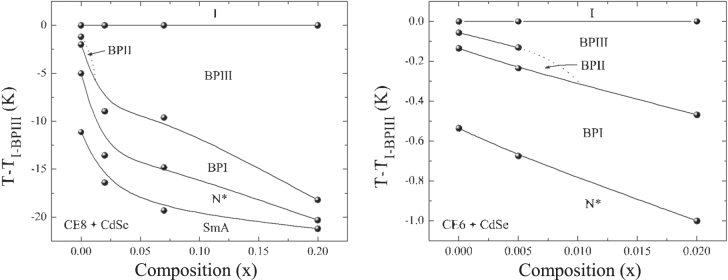
Phase diagrams for CE8 + CdSe mixtures (left) and CE6 + CdSe mixtures (right) [16]. Reprinted figure with permission from E Karatairi *et al* 2010 *Phys. Rev*. E **81** 041703, copyright 2010 American Physical Society.

For a mixture of CE8 and CdSe NPs, it was seen that at *x* = 0.02 (where *x* is the mass ratio of NPs and LC) provides an optimal phase widening of 20 °C. However, no significant phase widening is observed in the mixture of CE6 and CdSe NPs. For example, at *x* = 0.02 there was no evidence of BPIII phase, whilst *x* = 0.005 the presence of the BPIII phases was indicated (figure 7).

The mechanism that increases the temperature range of BPIII phase can be explained by the fact that, the disclination lines are almost entirely saturated with NPs so that it may achieve a wider temperature shift. In addition, NPs are possibly distributed non-uniformly inside the disclination lines, and stabilization of the shapeless BPIII type structure is more easily achieved, compared to the more ordered BPI and BPII structures. The proposed approach also suggests that the increase of NPs have a tendency to increase the disclination length within the structure, because the existence of NPs strongly decreases the condensation term. This process can be very useful for widening the BP temperature range, if NPs are strongly concentrated with disclination lines, which can be imposed by suitable surface treatment of NPs and their size [16].

#### 4.1.1. Electro-optical properties of NPs doped BPLC

In this section, we will discuss NPs doped BPLC within the context of electro-optical performance. Wong *et al* [67] studied the dispersion of gold nanorods (AuNRs) in a BPLC system and investigated their optical and thermal properties using various wt% of AuNRs. It was found that they expanded the BP temperature range by approximately 3.2 °C with 0.004% AuNRs. Later they have investigated the optical properties of 0.06% AuNRs dispersed BP samples, sandwiched in IPS cells of 5 *μ*m electrode spacing, line width and cell gap. Compared to switching voltage for the pure BPLC (49.4 V) and PSBPLC (∼140 V) the driving voltage of BPLC with dispersed AuNRs is low, at 41.9 V.

In a separate investigation, Hwang and Chien [99] reported a new technique to control the electro-optical properties of BP by adding aerosol NPs in a BPLC system. They reported that hydrophilic AG-d-BP samples reduced the switching voltage up to 64% compared to the pure BPLC. The switching voltage for 0.1% hydrophilic AG-d-BP mixture was 32 V compared 50 V for pure BPLC.

Wang *et al* [100] added another significant discovery in the topic of NP stabilized BPLCs where they dispersed a small amount of ZnS NPs in a BPLC system. Additionally, in a separate experiment Wang demonstrated a BPLC system with ferroelectric NPs [101]. In both reports, hysteresis-free electro-optical switching was observed. BPLC composites doped with ZnS NPs showed an increasing BPI range with the widest BP phase corresponding to 15.6 °C, and hysteresis-free switching with *V*_on_ of 75 V was achieved. In comparison, for ferroelectric NPs-doped BPLC system, Wang reported a BPI temperature range with a maximum BPI phase of 16.7 °C, and BPI doped with 0.7 wt% BaTiO_3_ NPs showed a hysteresis free switching with a driving voltage of 42 V. For reference, for both investigations the NP size and device architecture were comparable. The diameter of the ZnS NPs were ∼33 nm and BaTiO_3_ NPs were ∼30 nm. For both cases, an IPS cell with the following parameters: electrodes width 5 *μ*m, electrodes gap 5 *μ*m and cell gap 10 *μ*m, were used. The temperature range of BPI was extended, due to the suppression of the volume and free energy around the disclination core through the introduction of NPs in the BP system. Moreover, large dipole moments of the NPs enhanced the anchoring energy of LC molecules in the case of ZnS NPs, were able to significantly reduce the driving voltage [102, 103]. The ferroelectric NPs doping further improved the switching voltage through reduction of by more than 40% compared with ZnS NPs dispersed BPLC system due to the (1) a spontaneous strong polarization field (2) increasing dielectric constant and decreasing device parameter (such as electrode gap) which help to generate a uniform strong electric field followed by improving Kerr effect.

### 4.2. Polymer stabilization of BPs

**Figure 8.**
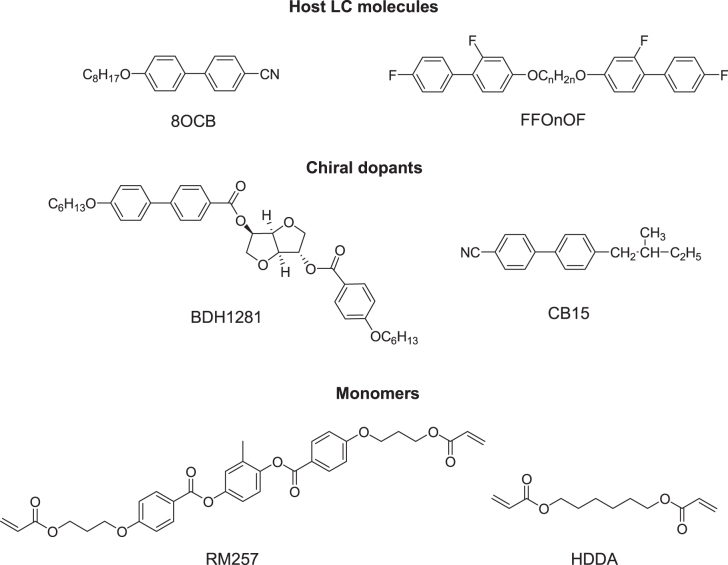
Examples of nematic host LC, chiral dopants, and monomers.

PSBPLCs demonstrate an acceptably wide mesogenic temperature window that includes the room temperature. In order to synthesize PSBPLC, a small amount of photoinitiator (∼0.5%) and monomers (∼8%) are added to the host LC (highest percentage of the system ∼86–92%) and chiral dopant (∼8–14%). Examples of host, chiral dopants and monomer structures are shown in figure 8.

#### 4.2.1. Characteristics of LC in the room temperature PSBPLC mixture

In the PSBPLC material system, the nematic LC forms the bulk of the mixture. Hence, it plays important role which influence the performance of the PSBPLC device [104] such as driving voltage, temperature range, and response time. To achieve a wide temperature range PSBPLC, a wide ranges nematic host LC is required [105–107]. Generally, the lower temperature limit is of less concern compared to the higher temperature limit, which is mainly defined by the isotropic to nematic clearing point of the LC host [108]. Moreover, the addition of monomers and photoiniatiors in the PSBPLC system has an inclination to lower the clearing temperature (nematic to isotropic temperature) compared to the pure nematic. Thus, a nematic LC host with a clearing temperature *T*_c_ > 80 °C is preferred.

When an electric field (*E*) is applied to a BPLC composite, the LC molecules reorient parallel to the electric field if *Δ∊* > 0, and perpendicular to the electric field if *Δ∊* < 0, where *Δ∊* is the dielectric anisotropy of the LC. The molecular reorientation of LC in electric field results in birefringence. In an IPS device, the on-state voltage (*V*_on_) is inversely proportional to the square-root of *K* [27]. The Kerr constant (*K*) of a BPLC is given by [28]:

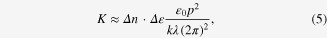
where, *k*, 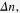 and 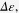 are the elastic constant, intrinsic birefringence, and dielectric anisotropy of the nematic host LC. The pitch length is denoted by 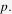 Equation (5) indicates that, a LC with high 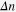 and large 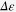 is extremely needed to increase the Kerr constant [109]. The response time of a PSBPLC material is given by [110]:

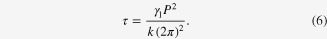


In the BPLC system *γ*_1_ is the rotational viscosity. This is intimately related to the chiral dopant and the host LC. To achieve fast response time a low viscosity host LC preferable [111]. Because from equation (6), response time (*τ*) is proportional to viscosity (*γ*) and inversely proportional to elastic constant (*k*). So as viscosity decreases and elastic constant increases, fast response time can be achieved [112].

The inclusion of chiral dopants induces double-twist in BPs [113]. BPs only appear as the chirality (*q*_0_ = 2*π*/*P*) exceeds a certain value. There are two ways to increase chirality of BPLC: (1) by increasing the concentration of chiral dopant or (2) by employing a chiral dopant containing a high helical twisting power (HTP) [114]. The melting point of chiral dopant is also an important consideration to select a chiral dopant. For an example, a very low meting point (∼4 °C) chiral dopant (CB15) is shown in figure 8. As a result, after mixing the chiral dopant with the host LC, the clearing temperature drops substantially as compared to the host LC. In order to widen the temperature range of BPLCs, a higher melting point chiral dopant is desirable whilst retaining good solubility [108].

**Figure 9.**
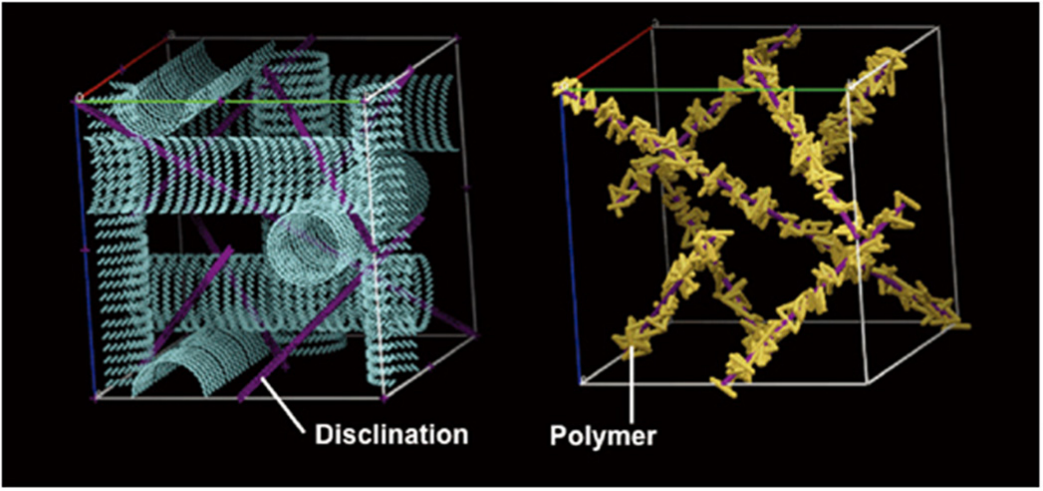
BPI stabilized by the polymer concentrated in the disclination lines [15]. Reprinted by permission from Macmillan Publishers Ltd: H Kikuchi *et al* 2002 *Nat. Mater.* **1** 64–8, copyright 2002.

The choice of monomer is essential in determining the stability of the BPLC [115–117]. Generally, a PSBPLC requires two types of polymer: bifunctional (e.g. RM257) and mono-functional (e.g. EHA or C12A) [118]. Polymer stabilized LCs describe systems in which a polymer network is formed within an anisotropic LC matrix. During the polymerization process a bi-continuous system is formed, where a continuous polymer network permeates a continuous LC phase shown in figure 9. The structure and order of the LC phase are transferred onto the polymer network, which thus mechanically stabilizes the phase it was formed in [119].

**Figure 10.**
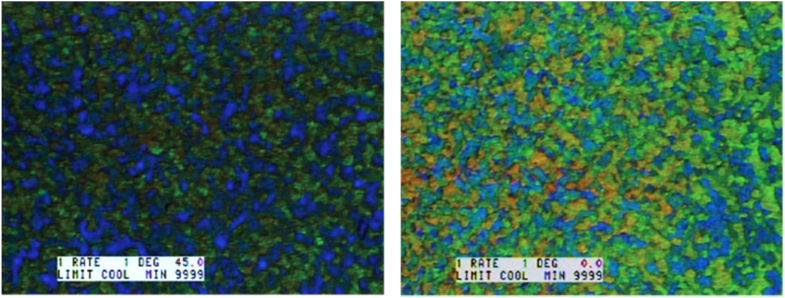
Polarizing optical microscopy image of PSBPLC. BPI at 46.3 °C (left) and BPI at 23.9 °C (right) [15]. Reprinted by permission from Macmillan Publishers Ltd: H Kikuchi *et al* 2002 *Nat. Mater.* **1** 64–8, copyright 2002.

By using a small fraction of polymer (8 wt%) in the overall BPLC mixture, a non-reactive BPLC can be stabilized as shown by Kikuchi *et al* in 2002 [15]. This was achieved by varying the percentage of the mono-functional monomers EH7 in the mixture. For example, an optimal condition of 1.6 mol% EHA results in a polymer stabilized BP range of >60 °C and the texture of this BP can be seen in figure 10.

In 2005, Coles and Pivenko also reported that, in dimer LCs with large flexoelectricity, the temperature range of the BP was more than 44 °C [88]. LC dimers are formed by linking two mesogenic groups by an alkyl chain spacer [120, 121]. Dimeric structures depend upon the number of carbon atoms in the spacer [122]. The nematic-isotropic transition temperature is found to alter as the number of carbon atoms in the alkyl spacer changes from odd to even [123]. Yoshizawa and co-workers reported that a mixture of T-shaped molecules and the binaphthyl derivative succeeded in forming the BP temperature ranges of 13 and 29 °C, respectively [124, 125]. Some other methods such as adding hydrogen-bond LCs have also been used to expand the BP temperature range [126]. The cross-linked polymer network is selectively concentrated in the disclination cores and the lattice structure of the BP is stabilized, thus extending the BPLC temperature range.

## 5. The response of the BPLCs to electric field

The influence of electric field on the structural manners of LC systems exhibiting BPs has been a topic of extensive experimental and theoretical attention. Interesting phenomena that have been investigated include the deformation of the BP unit cell, the reorientation of single crystals, and the total destruction of the three dimensional symmetry, resulting in a cholesteric structure [127]. Kerr-effect-induced isotropic-to-anisotropic transition in BPLCs has the possibility to become future generation display technology because it shows following four attractive features: (1) its response time is in the sub-millisecond range [33] which is almost ten times faster than the existing nematic LCDs; (2) it allows color-sequential display with a red, green, and blue light-emitting diode backlight, by this features the conventional pigment color filters can be eliminated; (3) does not involve any alignment layer to guide LC director to facilitate the fabrication process simplified; and (4) its viewing angle is wide and symmetric [128–130] because the voltage-off state is optically isotropic and voltage-on state forms multi-domain structures [54]. At the voltage-on state, although electric field induces birefringence and the LC index ellipsoid is elongated, the overall cubic symmetry of the BPLC is not changed. This again leads to a symmetric view of the LCD. Due to these features which are different from conventional nematic LCs, the iso-brightness contour of the IPS BPLC cell is more symmetric. However, two most important technical challenges need to be overcome: high operating voltage (∼50 *V*_rms_) and relatively low transmittance (∼65%), before widespread applications can take off [36].

Innovations in BPLC device structure for obtaining low voltage and high transmittance are driven by two main challenges: (1) high driving voltage; and (2) low transmittance [85]. The driving voltage of a PSBPLCD is directed by both device structure and Kerr constant of the employed material [54, 131]. From a material perspective, a large Kerr constant is desirable [132]. In recent times, a BPLC mixture with a large *K* ∼33.1 nm V^−2^ has been developed by Chen *et al* [133]. This new material will definitely speed up the emergence of BPLCDs. On the device viewpoint, several BP LCD structures such as protrusion electrodes [54, 134], wall-shaped electrodes, corrugated electrodes and double-penetrating fringe fields indicate a very positive trend to reduce the driving voltage [135–138] to the targeted 10 V. However, this is currently achieved with a sacrifice in transmission performance [139].

### 5.1. BPLC displays with corrugated electrodes

**Figure 11.**
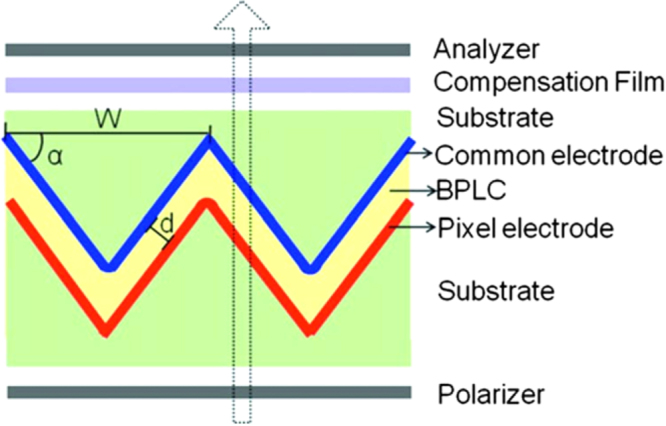
Schematic view of PSBPLCD architecture with corrugated electrodes [36]. Reprinted with permission from M Jiao *et al* 2010 *Appl. Phys. Lett.* **96** 011102. Copyright 2010, AIP Publishing LLC.

Jiao *et al* [36] proposed a low operating voltage (<10 V) and high transmittance (∼85.6%) PSBPLC display containing periodic corrugated electrodes as shown in figure 11. This device architecture creates a strong horizontal electric field component, and more importantly this field penetrates deeply into the LC medium meaning jointly these two factors contribute to lower the driving voltage to ∼9.9 *V*_rms._ The rms value is the square root of the mean (average) value of the squared function of the instantaneous values. The term rms, refers to time-varying sinusoidal voltages, currents or complex waveforms yields the magnitude of the voltage over one period of the sinusoidal wave. Simultaneously, the electric field created by such an arrangement is uniformly circulated across the entire pixel area and by this can obtain high transmittance (∼85%). To broaden the viewing angle a biaxial compensation film has been used. As compared to typical dimensions of patterned IPS electrodes, the electrode width (*W*) is quite large in this device (∼40 *μ*m). The top substrate is coated with common electrode (without patterning) whilst bottom substrate is coated with pixel electrodes. Furthermore, a BPLC material with *K* ∼ 12.7 nm V^−2^ is assumed.

**Figure 12.**
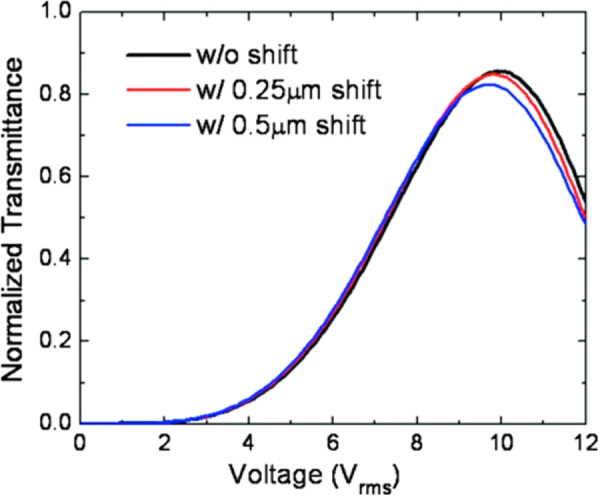
Normalized *V*–*T* curves of the PSBPLCD with corrugated electrodes [36]. Reprinted with permission from M Jiao *et al* 2010 *Appl. Phys. Lett.* **96** 011102. Copyright 2010, AIP Publishing LLC..

Jiao *et al* investigated voltage-dependent transmittance (*V*–*T*) curves of the BPLC device at *λ* = 550 nm, with variation in misalignment between the top and bottom electrodes, as shown by figure 12. For an ideal case, the switching occurs at ∼9.9 *V*_rms_ and the highest transmittance reached 85.6% [36].

When the misalignment i.e. the horizontal alignment difference between top and bottom electrodes was 0.25 *μ*m, the change in *V*–*T* curve was negligible. By increasing the misalignment to 0.50 *μ*m, *V*_on_ fell to ∼9.7 *V*_rms_ and transmittance reached 82.4%. From figure 12, the *V*–*T* curves overlap with each other when *V* < 9 *V*_rms_, which means the device is insensitive to horizontal shift if it was driven lower than 9 *V*_rms_, where the transmittance was maintained at ∼80%. Other parameter variations, such as enhancing the inclination angle of electrodes or a thinner cell gap are also effective in reducing the driving voltage. For example, by an increased inclination angle, the horizontal component of the induced birefringence was enhanced and the effective path length of incident light is increased, thus with a lower voltage, expected phase retardation can be achieved. Furthermore, when the cell gap decreased, the optical path length *d*_opl_ is decreased; but on the other hand the induced birefringence (*Δn*) increased in quadratic manner due to the stronger electric field. Summarily, since phase retardation is proportional to *d*_opl_*Δn*_nind_, a lower driving voltage can result in the same phase retardation in a thinner cell gap. Moreover, the dead zones become narrower in a thinner cell and thus transmittance will be improved [36]. Also by increasing the electrode width, transmittance can be increased. As the electrode width increased, the dead zones decreased because dead zones were found at every turning edge area. Thus, the induced birefringence along the vertical direction at every turning edge will be low as it makes no contribution to phase retardation, resulting in high transmittance.

### 5.2. Protrusion electrodes for BPLC devices

**Figure 13.**
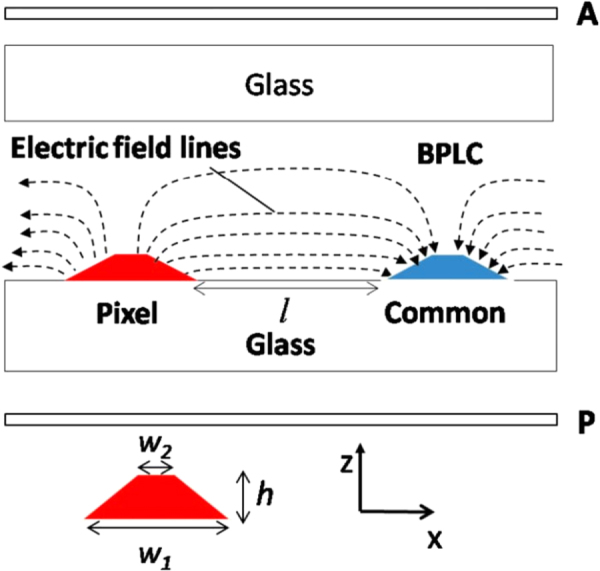
Cross section view of the BPLCD with protrusion electrodes [54]. Reprinted with permission from L Rao *et al* 2009 *Appl. Phys. Lett.* **95** 231101. Copyright 2009, AIP Publishing LLC.

**Figure 14.**
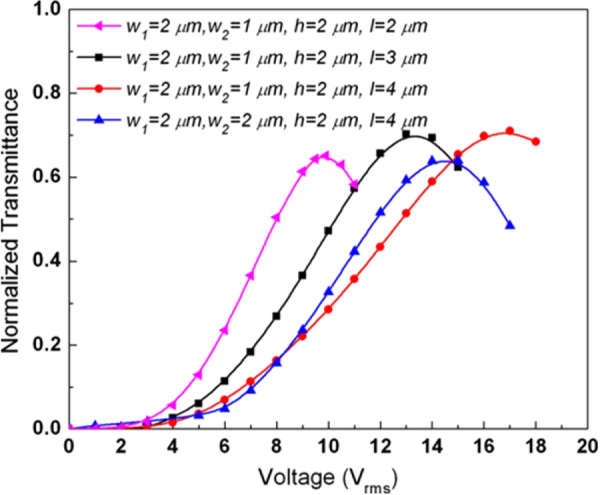
Normalized *V*–*T* graphs of the BPLCD for protrusion electrodes with different electrode dimensions [54]. Reprinted with permission from L Rao *et al* 2009 *Appl. Phys. Lett.* **95** 231101. Copyright 2009, AIP Publishing LLC.

Rao *et al* reported a BPLC cell with protrusion electrode structure shown in figure 13, where the two electrodes (common and pixel) form a trapezoid shape [54] of following dimensions: bottom width (*w*_1_), top width (*w*_2_), height (*h*) and space (*l*) between electrodes. This electrodes structure, not only generates strong electric field between the pixel and common electrodes (horizontally) but also penetrate deeply into the LC bulk region (in a vertical direction) which plays significant role to reduce operating voltage. This type of device structures have been widely used in multi-domain vertical alignment mode LCDs for capturing wide viewing angles. Thus, the fabrication process of protrusion electrodes (by coating ITO on top of protrusions) is not a technical hurdle. A typical protrusion electrode with *w*_1_ = 2 *μ*m, *w*_2_ = 1 *μ*m, *h* = 2 *μ*m, and *l* = 4 *μ*m is shown in figure 14, the highest transmittance obtained with this structure is ∼71% at 17 *V*_rms_ [54].

Decreasing the spacing width *l*, will reduce the on-state voltage, due to the strong lateral electric fields. However, this is compromised by a decrease in transmission. When the top of trapezoid *w*_2_ increased in length this resulted in a more uniform lateral electric field, again at the cost of lower transmittance. Likewise, a larger taper angle will have a similar effect. The low transmittance was exacerbated by the presence of a ‘dead zone’, i.e. a region above the electrodes where the vertical field does not contribute to transmittance. One solution was to increase the height of the electrode in order to minimize the ‘dead zone’, but for higher electrode profiles (*h* > 4 *μ*m) the fabrication process becomes more difficult [54].

#### 5.2.1. Enhanced protrusion electrodes for BPLC device

**Figure 15.**
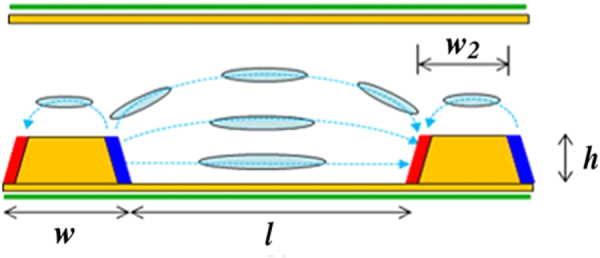
BPLCD with enhanced protrusion electrodes [139]. © 2011 IEEE. Reprinted, with permission, from Y Li and S T Wu 2011 *J. Display Technol.* **7** 359–61.

An enhanced protrusion electrode was proposed by Li *et al* [139] for displays which increased transmittance whilst maintaining a low driving voltage. The structure of the enhanced protrusion electrode architecture is shown in figure 15 where two different electrodes are coated on each side of a protrusion such as one side-wall of a protrusion is coated with pixel electrode, and the other is with common electrode. The electric field distribution between protrusions is similar to the conventional protruded structure, thus low operating voltage is still maintained. On the other hand, dead zone on the top of the protrusions was eliminated, because of the IPS-like arrangement on the top of the protrusions was maintained. This is similar to an IPS configuration, it generated horizontal fields and induced transmittance. As a result, the total transmittance was amplified by elimination of the dead zone which exists in normal protrusion electrodes.

**Figure 16.**
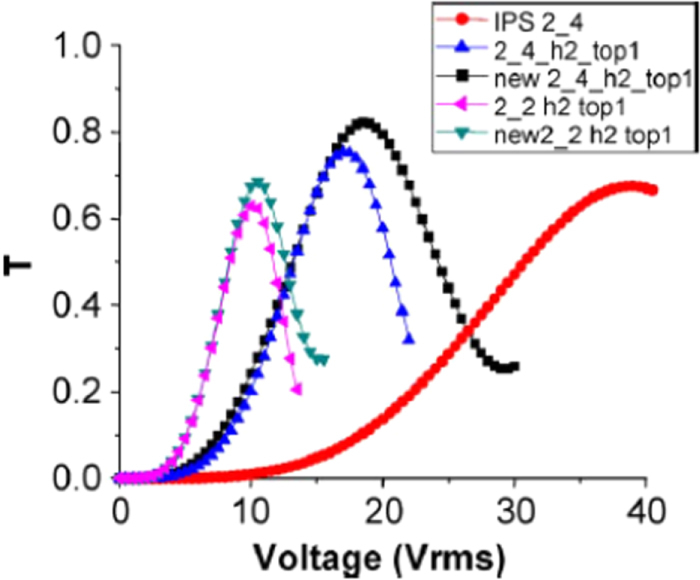
*V–T* curves of the BPLC for conventional protrusion structures versus new protrusion structures [139]. © 2011 IEEE. Reprinted, with permission, from Y Li and S T Wu 2011 *J. Display Technol.* **7** 359–61.

A comparison of the transmission of enhanced protrusion electrodes with normal protrusion electrodes can be seen in figure 16.

### 5.3. Double sided IPS electrodes for BPLC devices

**Figure 17.**
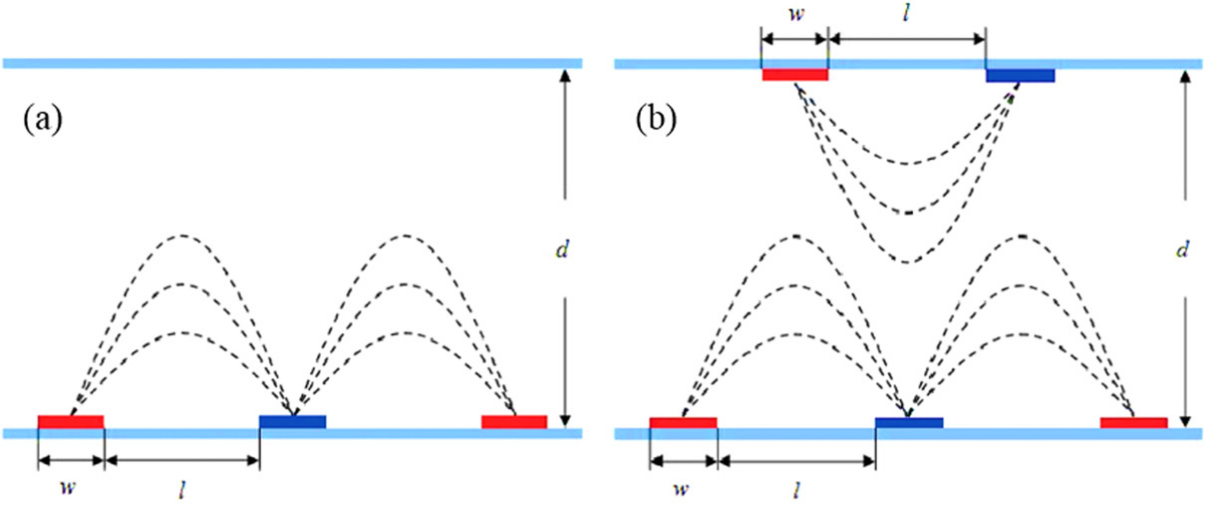
BPLCD with (a) single side IPS architecture and (b) double side IPS architecture [44]. Reprinted with permission from Y Chen *et al* 2011 *Liq. Cryst.* **38** 555–9.

A conventional IPS structure is shown in figure 17(a) where only in the bottom of the substrate of the IPS electrodes were etched thus creating a lateral electric field. Here, width, electrode gap and cell gap are represented by *w*, *l* and *d* respectively. In comparison, a double sided IPS architecture was shown in figure 17(b), reported by Chen *et al* where in the top of the substrate the IPS electrodes are also etched and placed correspondingly with the center of the strip electrode gap on the bottom substrate. It is evident that alternating position of the top and bottom electrodes in DS-IPS electrodes can induce more horizontal fields than conventional IPS electrodes [44]. The index of refraction here is higher than that of the typical IPS architecture; therefore, BPLCD driven by double side IPS electrodes has a low operating voltage.

**Figure 18.**
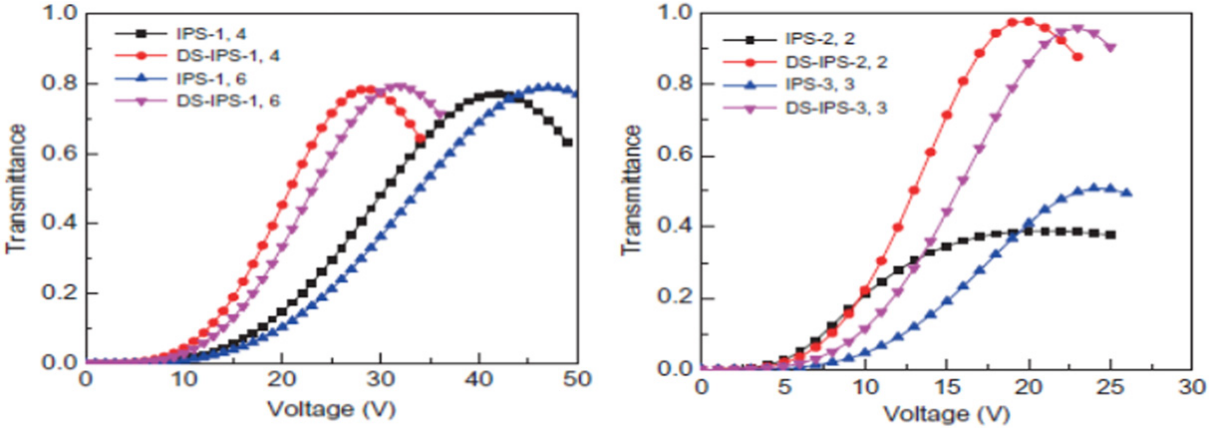
Voltage and transmission comparison between IPS and DS-IPS structure [44]. Reprinted figure with permission from Y Chen *et al* 2011 *Liq. Cryst.* **38** 555–9.

A comparison of the performance between DS-IPS electrodes and conventional IPS electrodes for BPLCD is shown in figures 18(a) and (b).

For example, for following electrode dimensions *w* = 1 *μ*m, *l* = 4 *μ*m, and *d* = 10 *μ*m (IPS-1,4) the DS-IPS has an operating voltage of 25 V, compared to 36 V for conventional IPS. The transmission is also slightly improved for the DS-IPS electrodes cell. The observation of both reduced driving fields and improved transmittance is directly attributable to the DS-IPS electrode structure, which significantly increases the constant strong horizontal electric fields in the entire area. This improvement is even more evident when comparing the IPS-2,2 structure with the DS-IPS-2,2 (see in figure 18), where the driving voltage is reduced 21–19.5 V and transmittance is increased from ∼39.1 to ∼98% [44]. As is evident from this section, clever design of electrodes can significantly improve the performance characteristics of a BPLC display.

### 5.4. VFS

**Figure 19.**
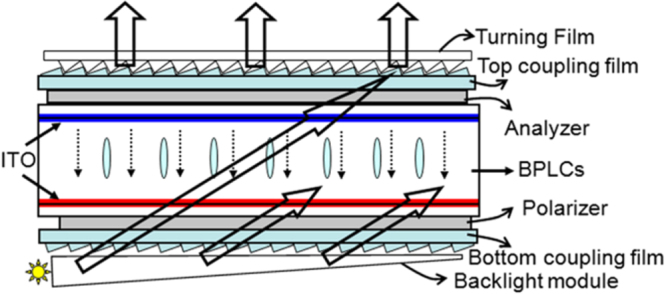
Device architecture of VFS mode BPLCD [55]. Reprinted with permission from H C Cheng *et al* 2011 *Appl. Phys. Lett.* **98** 261102. Copyright 2011, AIP Publishing LLC.

IPS structure is very popular in current display technology. However, in IPS mode BPLC devices, the electric field drives the BPLC molecules in lateral direction. In such mechanism, a BPLC with positive dielectric anisotropy induced birefringence along the electric field. One of the main drawback of this mode is, the electric fields are held firmly close to the electrodes edge rather than penetrate deeply and uniformly into the BPLC bulk which in turn causes lattice deformation. Due to this lattice deformation and less phase retardation the hysteresis and driving voltage is high in IPS mode BPLC devices. To overcome this issues Cheng *et al* [55] demonstrated a VFS mode BPLC device where the electric field is in opposite direction of IPS mode and the electric field uniform spatially. He reported that in a longitudinal direction of electric field, large phase retardation can be achieved by controlling the oblique angle through the cell gap. He also presented a hysteresis free BPLC device with low driving voltage (∼10 V). The structure of the VFS device is shown in figure 19. Furthermore, VFS mode BPLC device achieved approximately twofold improvement in response time and 100% optical efficiency due to its vertical and uniform electric field. The Kerr effect states that induced birefringence is proportional to *E*^2^. Thus, as cell gap decreases the induced birefringence becomes large. However, the decrease in cell gap also decreases the beam path. The tradeoff between the induced birefringence and beam path requires optimization of the cell gap in order to achieve a comparison between these two parameters. Thus, an optimized cell gap and incident angle could produce large phase retardation whilst keeping small induced birefringence and minimize the operating voltage.

### 5.5. Methods for enlarging the Kerr constant

Reduction in operating voltage may be achieved by an alternative approach, through the development of a BPLC material with a large Kerr constant [140]. Most of the BPLC materials developed until now possess a K value ranging from 0.4 to 4 nm V^−2^ [132] and the corresponding driving voltage is over 100 V, which is almost impossible to operate using amorphous silicon thin-film transistors (a-Si TFTs).

BPLC materials with larger Kerr constant are urgently needed to develop lower driving voltage BPLCs. Iwata *et al* reported a mechanism for enlarging the Kerr constant [141]. Equation (4) can be written as

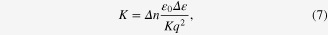
where *k* and 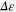 are the elastic constant and the dielectric anisotropy of the LC materials respectively, the elastic deformation wave number denoted by *q*, which is in inversely proportion to the lattice constant *a* of a BP; the cholesteric pitch (*P*_0_) of LC materials is related to *a*. For instance, in the case of BPI *P*_0_ is almost equal to *a.* By increasing the *a,* Kerr constant can be increased. However, the Bragg diffraction of the visible wavelength BP is colored, therefore, increasing the above its certain value will lead lower contrast ratio of displaying image. Thus, there is a crucial trade-off between the operating voltage and the contrast [141]. Rao *et al* [142] reported a BPLC material, designated as JC-BP01M, which exhibits a Kerr constant *K* ∼13.7 nm V^−2^ at 20 °C. If JC-BP01M is employed in a cell with protruded electrodes [54], the driving voltage would drop to below 10 V. This will qualify BPLC to be operated by a-Si TFTs which will accelerate the development of BPLC for display and photonic switching applications [143]. Later, Wittek *et al* proposed a new material for PSBPLC which improved the chemical stability and operating voltage [144]. Haseba *et al* reported a reduction in the operating voltage and increment in Kerr constant by increasing the product of birefringence and dielectric anisotropy of the host LC material [145]. However, there is a trade-off between the high dielectric anisotropy and optical efficiency since a large *Δ∊* required longer charging time to reach the desire voltage. This charging problem was later improved by Tu *et al* by using bootstrapping method [146]. Finally, Haseba reported a PSBPLC material with large product of *Δn* × *Δ∊* where the operating voltage is 23 V. In 2013, Chen *et al* reported a BPLC material named JC-BP06N, whose Kerr constant is 33.1 nm V^−2^, which is 2.2 times larger than JC-BP01M. A low driving voltage 8.4 V with 0.9% hysteresis was achieved using JC-BP06N in VFS cell [133].

## 6. Electro-optical properties of BPLC depending on polymer and NPs stabilization

Electro-optical properties such as operating voltage, transmittance and hysteresis of BPLC devices based on electrodes structure, material properties, polymer network and NPs doping will be compared in this section.

### 6.1. Electrodes structure and Kerr constant effect on optical properties of PSBPLC display devices

In the previous section, experimental and simulation approaches regarding device structures for BPLC displays are discussed. In addition, in this section, their electro-optical properties (Kerr constant, dielectric anisotropy and birefringence) and device structure are summarized in table 3 and discussed.

**Table 3.** A comparison of electro-optical properties for different device structures.

		Materials				Performance	
Electrode structure		Name	Kerr constant (*K*) (nm V^−2^)	Dielectric anisotropy (*Δ∊*)	Birefringence (*Δn*)	Switching voltage (*V*)	Transmittance (%)
(A)	Corrugated	Simulated	12.7	32	0.30	∼9.9	85.6
(B)	Protrusion	Simulated	Not found	Not found	Not found	∼10	∼70
(C)	Enhanced protrusion	JC-BP01M	13	94	0.17	∼10	∼69
(D)	Double sided IPS	Simulated	12.7	32	0.30	∼19.5	98
(E)	VFS	JC-BP01M	13	94	0.17	∼16	100
(F)	VFS	JC-BP06N	20.9	470	0.17	8.4	100

From the materials view point, the Kerr constant played a main role to reducing the switching voltage as concluded from table 3. JC-BP06N has the highest Kerr constant and shows very large dielectric anisotropy value which reduces the driving voltage to 8.4 V for case F, when the material was employed in the VFS cell. Similarly, for materials with a comparable Kerr constant (12.7–13.0 nm V^−2^ for cases A, C, D and E) showed the best improvement in operating voltage (9.9 V) when employed in the case of a corrugated cell (case A). On the other hand, from the device view point there is a trade-off between the driving voltage and transmittance for every electrode structure, except for the VFS cell (cases E and F) where 100% transmittance was achieved for a relatively low driving voltage. After the VFS cell, the DS-IPS cell also offered high transmittance (98%); however, the driving voltage was relatively high (19.5 V). Comparing the VFS cell (case E and F) with the enhanced protrusion cell (case C), the VFS cell provided high transmittance whilst the enhanced protrusion device offered a low on-stage voltage. Furthermore, the corrugated device showed better performance in terms of driving voltage (9.9 V) compared to the DS-IPS cell, but again the trade-off is in the transmittance level.

### 6.2. Electro-optical properties of BPLC under polymer stabilization or NP doping

Stabilization of the BP is achieved through either polymer or NP dispersion in the BP disclination lines. They do however result in differing electro-optical performance. For NPs doped systems, the lowest driving voltage reported was 32 V using aerosol NPs. On the other hand, a hysteresis free BPLC device was achieved by doping ferroelectric NPs, but the driving voltage was comparatively high i.e. 42 V. In comparison, as reviewed in section 6.1, PSBPLC devices have demonstrated superior electro-optical performance, i.e. being able to render 100% transmittance and hysteresis free electro-optical switching at voltages as low as 8.4 V for the VFS mode device. It is recommended that a large Kerr constant and effective electrode structure should be developed in devices based on NPs doped BPLC system, in order to render them feasible for next generation BPLC display applications.

## 7. Recent developments in amorphous BP III LC

Unlike BPI and BPII, BPIII does not exhibit Bragg reflections and has no three-dimensional orientational long range order. However, there is certain correlation of microscopic molecular orientation in the order of the lattice size between BPIII and BPII. Due to amorphous and complex structure to date, limited work has been done on BPIII, the structure and a definite model of BPIII is yet to be conclusively understood, despite a few reports and models proposed [147–149]. In 2011, Henrich *et al* [150] proposed through simulation that BPIII is an amorphous continuum of disclination lines and that these disclination lines are kinetically and thermodynamically stabilized over crystalline BPs at intermediate chiralities. He also suggested that in the presence of an applied electric field this amorphous network becomes ordered.

Later, a few approaches were reported to stabilizing the BPIII phase, such as (1) doping with NPs, which extended the temperature by approximately 20 °C, (2) doping an achiral bent-core system with chiral dopant which stabilized the system over 20 °C due to the presence of smectic clusters [151], (3) utilization of novel chiral T-shaped compound which exhibited BPIII with a temperature range of 13 °C and was assumed to be stabilized by the coupling between molecular biaxiality and chirality [124], (4) design of a chiral T-shaped compound which possess a flexible spacer which was found to exhibit a room temperature BPIII with a temperature range of about 30 °C on cooling [152], (5) UV irradiation of a bent-core LC bearing an azo linkage doped with chiral molecules BPIII resulted in stabilization over 20 °C [153], (6) design of a T-shaped compound named ‘BP stabilizer’ which added to a nematic mixture and transformed to BPIII with 7.2 °C window by adding large HTP chiral dopant [154] and (7) use of a polymer network where the structure of BPIII was stabilized and the temperature range was widened by more than 10 °C [155].

**Figure 20.**
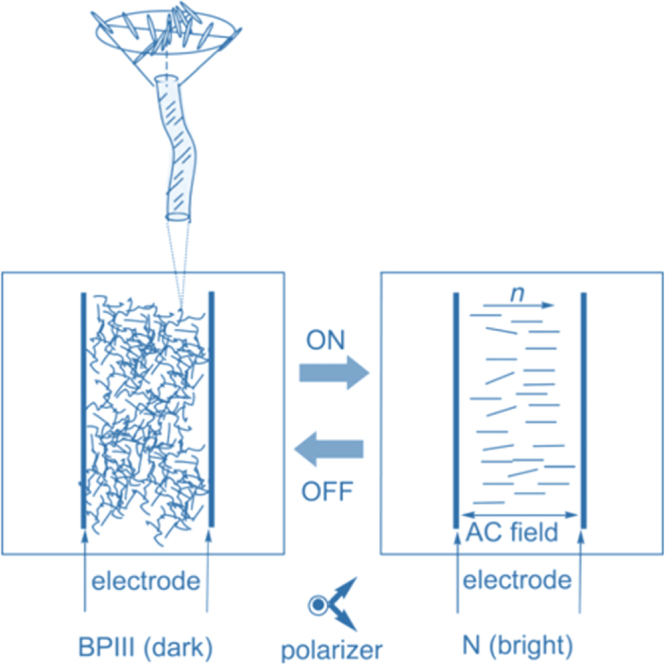
Electro-optical switching in BPIII proposed by Yoshizawa [158]. Reproduced with permission from A Yoshizawa 2008 *J. Soc. Inf. Disp.* **16** 1189–94. Copyright 2008, John Wiley and Sons.

Besides thermal stability, electo-optical properties of BPIII was investigated [156–161] due to the following advantages over cubic BP, i.e. (a) hysteresis free characteristics, (b) small residual birefringence, (c) lower threshold and saturated voltages, and (d) higher stability against an electric field [162]. An IPS cell with BPIII sample was demonstrated by Yoshizawa *et al* [154]. However, the driving voltage was comparatively high, i.e. 140 V compared to the typical switching voltage for the BPI phase. On the other hand, the device showed hysteresis free *V*–*T* curve with submillisecond switching (rise time 0.4 ms and decay time 0.8 ms). Additionally, a possible switching mechanism of BPIII is shown in figure 20, which describes the electric field induced phase transition between BPIII and N. It is proposed that BPIII has a macroscopically isotropic order but microscopic TN order. On the application of an electric field amplifies the nanoscale twist of the BPIII into the micro scale of the nematic organization, which is observed in a change in birefringence. Removal of the electric field restores the double-twist structure of BPIII. Using a conventional IPS cell, fast switching (∼0.9 ms) and hysteresis free polymer stabilized BPIII device was reported by Chou *et al* [155]. Recently, a BPIII device with 3 ms response time was described by Chen and co-worker [163]. Based on their results, they proposed that two other effects which dominate the electro-optical properties of BPIII besides Kerr effect, which are flexoelectricity and local dielectric couplings. The entire mechanism of the switching process in the BPIII cell is not clear. However, the operating voltage and temperature stability are expected to be improved through material or device architecture in order to be used in next generation display and photonic applications.

## 8. Flexible displays

BPLCs are not only attractive for flat plane displays, but are also potential candidates for next-generation flexible display materials owing to the self-assembled structure, which eliminates the surface alignment layer and alignment processing like rubbing, and sub-millisecond electro-optical response time. For flexible display technology, following features should be considered; shock resistance, light weight, thinness, and film substrates over glass substrates. The main drawback of conventional LCD is, polyimides layer on glass substrate which required to align the LC molecules and which need to be process at high temperature. This thermal process is incompatible with plastic substrate because the structure and uniformity might be change during heating process.

Thus, alignment free BPLC is very appropriate for such flexible display devices [164]. However, Kimura *et al* demonstrated a PSBPLC display on bendable single polyethylene terephthalate substrate but the BP temperature range was ∼5.2 °C and driving voltage was ∼45 V [165]. On the other hand, Castles *et al* reported a room temperature stretchable BPLC by increasing the percentage of reactive monomers and UV exposure intensity which broaden the capabilities of BPLC in flexible and photonic devices [166].

## 9. Other potential applications

For the photonic applications electric field tunable BPLCs are very promising, given its fast response time [167]. Tunable color devices [168] are demonstrated experimentally by using the electrostriction effect [88, 169, 170]. For example, continuous tuning of wavelengths have been achieved in the range of 572–506 nm, with tens of millisecond decay time range [169]. For the reflective display [171, 172] applications and optical filter devices, colors that can be tuned electrically are desirable. BP applications can also be found in lasing [173–175]. BPLC, doped with little amount of dye, can emit light when excited inside a BP lasing device [176]. The color change in PSBPLC is not as obvious as in pure BPs, due to the negligible lattice distortion in the electric field. Even so, the refractive index variation due to Kerr effect can also be employed in photonic applications, such as tunable phase gratings [177] and tunable micro-lenses [178–180]. BPs are also recognized as the model systems for artificial photonic band gap materials or natural photonic crystals due to the recent interest in photonic crystals [181, 182].

## 10. Conclusions

In applications where fast response time are required, such as high definition displays and photonics switching, BPLCs are attractive candidate materials. However commercial realization of BPLC involves overcoming major challenges such as high driving voltage and low transmittance. The narrow temperature range (0.5–1 °C) of BP has been dramatically improved through incorporation of NPs or mixing with polymers. On the device level, operating parameters such as high switching voltage, hysteresis and low transmittance need to be overcome. Novel BPLCD device architectures, especially in terms of electrode design, have shown significant improvements in these three parameters, bringing BPLC devices closer to the targeted operating voltage (<10 V), low hysteresis and high transmittance (>90%). Coupling the efforts in material development and device designs implies that great improvements in BPLC devices have been achieved in recent years. Thus the realization of practical BPLC devices especially in the realm of displays technology is becoming closer to fruition.
